# Pulsed field ablation vs high-power short duration/very high-power short duration pulmonary vein isolation—Systematic review and meta-analysis with Kaplan-Meier reconstructed individual patient data

**DOI:** 10.1016/j.hroo.2025.06.011

**Published:** 2025-08-05

**Authors:** Raymond Pranata, William Kamarullah, Giky Karwiky, Chaerul Achmad, Mohammad Iqbal, Jae-Sun Uhm

**Affiliations:** 1 Department of Cardiology and Vascular Medicine, Faculty of Medicine, Universitas Padjadjaran, Hasan Sadikin General Hospital, Bandung, Indonesia; 2 Division of Cardiology, Department of Internal Medicine, Severance Hospital, Yonsei University College of Medicine, Seoul, Korea

**Keywords:** Atrial fibrillation, Pulsed field ablation, High-power short duration ablation, Very high-power short duration ablation, Pulmonary vein isolation

## Abstract

**Background:**

High-power/very high-power short-duration (HPSD/VHPSD) pulmonary vein isolation has greater efficacy than does conventional pulmonary vein isolation, while pulsed field ablation (PFA) is associated with a significantly shorter procedural duration with comparable efficacy.

**Objective:**

This meta-analysis compared the efficacy, procedural duration, and safety of PFA vs HPSD/VHPSD ablation.

**Methods:**

Atrial tachyarrhythmia (ATa) recurrence was defined as any episode of atrial fibrillation, flutter, or tachycardia lasting >30 seconds, detected by Holter monitoring or electrocardiography, after a 3-month blanking period. The intervention group received PFA and the control group received HPSD/VHPSD ablation.

**Results:**

Eight studies (mostly retrospective observational) comprising 1411 patients were analyzed. ATa recurrence occurred less frequently in the PFA group than in the HPSD/VHPSD ablation group (15% in PFA vs 25% in HPSD/VHPSD ablation; odds ratio 0.57; 95% confidence interval [CI] 0.40–0.81; *P* = .002; I^2^ = 31.4%, *P* = .374). Meta-regression analysis indicated that the benefit of PFA was not significantly influenced by paroxysmal atrial fibrillation, sex, age, hypertension, or left ventricular ejection fraction. Reconstructed Kaplan-Meier curves showed greater freedom from ATa recurrence in the PFA group, and subsequent reconstructed individual patient data Cox regression analysis showed a lower hazard ratio (hazard ratio 0.68; 95% CI 0.48–0.97; *P* = .033). Pulmonary vein reconnection rates were comparable (32% in PFA vs 35% in HPSD/VHPSD ablation; odds ratio 0.84; 95% CI 0.53–1.34; *P* = .473). PFA significantly reduced total procedural duration (mean difference –29.67 minutes; 95% CI –38.31 to –21.03 minutes; *P* < .001). Complications rates were similar between the 2 groups.

**Conclusion:**

PFA was potentially associated with a comparable or reduced ATa recurrence rate and a shorter procedural duration compared with HPSD/VHPSD ablation while maintaining similar complication rates. Further randomized controlled trials are warranted to validate these findings.

**PROSPERO registration number:**

CRD420251034443.

**Key Findings**

▪ Pulsed field ablation (PFA) was potentially associated with a comparable or reduced rate of atrial tachyarrhythmia (ATa) compared with high-power/very high-power short-duration (HPSD/VHPSD) ablation, as demonstrated by pooled binary outcomes, reconstructed individual patient data regression, and survival analysis.
▪ The benefit of PFA in reducing ATa recurrence was not significantly influenced by the presence of paroxysmal atrial fibrillation, sex, age, hypertension, or left ventricular ejection fraction.
▪ There were no significant differences in the number of repeat ablation procedures or in the incidence of pulmonary vein reconnections during repeat interventions.
▪ Although the total procedure time was shorter in the PFA group, fluoroscopy time was longer compared with the HPSD/VHPSD ablation group.
▪ Complications rates were similar between the PFA and HPSD/VHPSD ablation groups.

## Introduction

Atrial fibrillation (AF) is the most common sustained arrhythmia, and catheter ablation is increasingly being used as a first-line therapy, particularly in cases of paroxysmal AF.^1^ Recent advancements in ablation technology have improved arrhythmia-free survival, especially for patients with paroxysmal AF.^2^^,^^3^ However, the long duration of ablation procedures often results in longer wait times, which, in turn, prolongs the diagnosis-to-ablation time. This delay has been linked to increased morbidity and a higher rate of atrial tachyarrhythmia (ATa) recurrence.^4^, ^5^, ^6^ Therefore, it is crucial to develop ablation strategies that combine high efficacy with streamlined workflows and reduced procedural duration.

High-power/very high-power short-duration (HPSD/VHPSD) pulmonary vein isolation (PVI) has demonstrated greater efficacy and shorter procedural duration than does conventional PVI.^2^ Pulsed field ablation (PFA) has also been shown to significantly reduce procedural duration while maintaining efficacy comparable to that of thermal ablation.^3^ Both techniques offer the potential to perform PVI with reduced procedure times and comparable, if not superior, efficacy relative to conventional thermal ablation, which has been the standard approach for decades. This systematic review and meta-analysis aims to compare the efficacy, procedural duration, and safety of PFA vs HPSD/VHPSD ablation, incorporating Kaplan-Meier reconstructed individual patient data (IPD) and meta-regression analyses.

## Methods

### Protocol and registration

This systematic review was conducted in accordance with the guidelines outlined in the *Cochrane Handbook for Systematic Reviews of Interventions* and reported following the Preferred Reporting Items for Systematic Reviews and Meta-Analyses (PRISMA) framework. The review protocol was registered on PROSPERO (CRD420251034443).

### Literature search strategy

**Figure 1:**
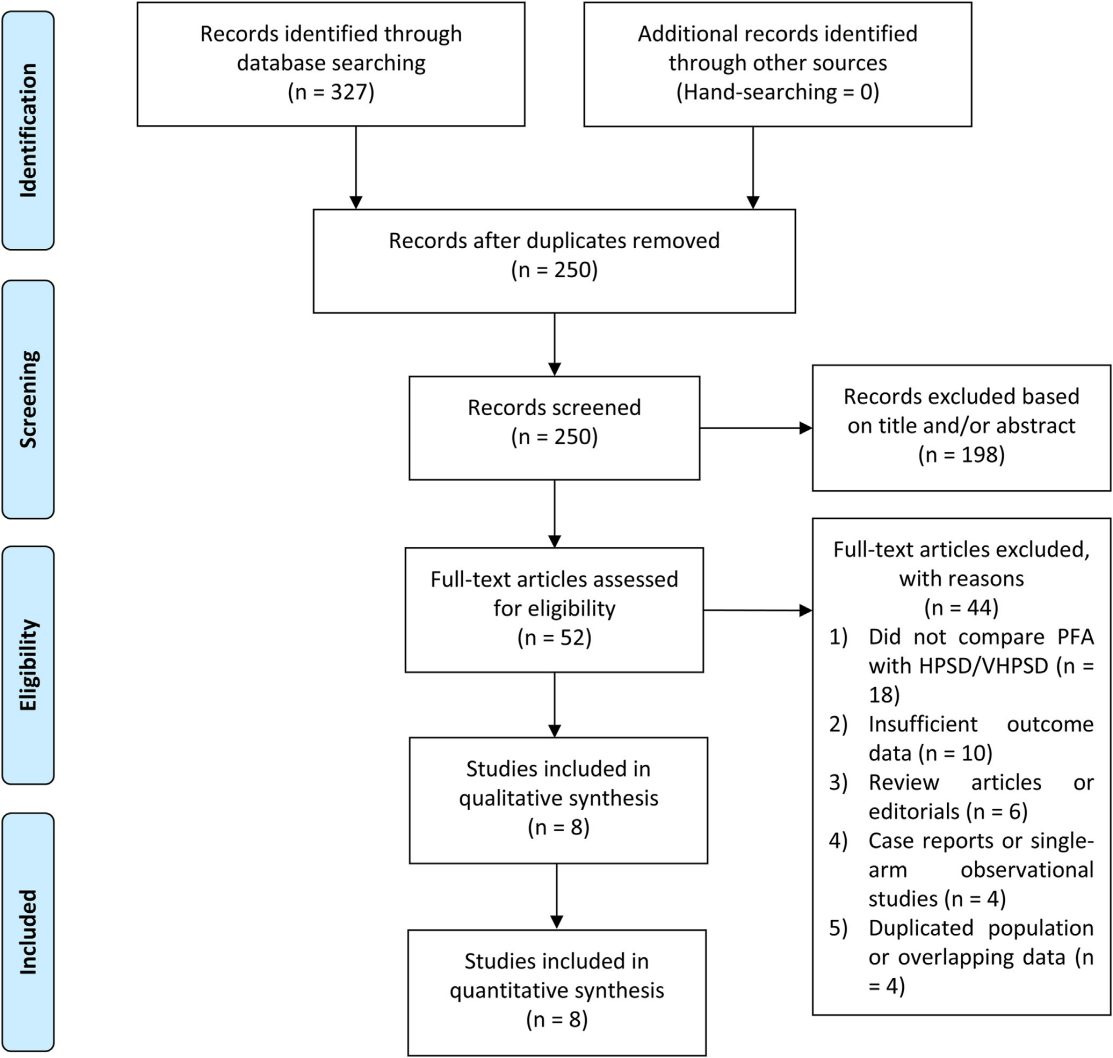
Preferred Reporting Items for Systematic Reviews and Meta-Analyses (PRISMA) flowchart. HPSD = high-power short duration; PFA = pulsed field ablation; VHPSD = very high-power short duration.

A comprehensive literature search was conducted across PubMed, SCOPUS, Europe PMC, and ScienceDirect databases up to April 16, 2025. The search terms included the following: (“Pulsed Field Ablation” OR “PFA”) AND (“High-power Short Duration” OR “HPSD” OR “Very High-power Short Duration” OR “vHPSD”) AND (“Pulmonary Vein Isolation” OR “PVI” OR “ablation”) AND (“Atrial Fibrillation”). Search strategies were customized to meet the specific syntax and requirements of each database. This search process adhered to PRISMA guidelines, and a flow diagram (Figure 1) illustrates the steps involved in article selection and screening.

### Study selection, inclusion, and exclusion criteria

This study included patients with either paroxysmal or nonparoxysmal AF who underwent either an initial or repeat AF ablation procedure, regardless of whether additional ablation beyond PVI was performed. Participants were categorized into 2 groups on the basis of the ablation technique used: those receiving PFA as the intervention group and those receiving HPSD/VHPSD ablation as the control group.

The primary outcome was *ATa recurrence*, including AF, atrial flutter, and atrial tachycardia, defined as any episode lasting longer than 30 seconds and occurring after a minimum of a 3-month postprocedure blanking period. Secondary outcomes included the rates of repeat ablation and pulmonary vein reconnection, total procedure duration, fluoroscopy time, and complication rate.

This study included randomized controlled trials, as well as both prospective and retrospective observational studies, that directly compared PFA with HPSD/VHPSD ablation with regard to the outcomes of interest. Eligible publications included full-text articles, conference abstracts, and research letters. Studies with insufficient data were excluded. In addition, reviews, editorials, case reports, commentaries, and animal studies were excluded from this systematic review.

### Data extraction and risk of bias assessment

Two reviewers independently extracted data using a standardized form that captured the characteristics of each included study, such as participant age, study design, sample size, inclusion criteria, presence of paroxysmal AF, left atrial (LA) diameter, left ventricular ejection fraction (LVEF), follow-up duration, repeat ablation rate, pulmonary vein reconnection rate, procedure time, fluoroscopy time, and complication rate.

To assess the risk of bias in observational studies, the Newcastle-Ottawa Scale was used. Studies scoring ≥7 were classified as low risk, while those scoring ≤6 were considered high risk. Disagreements between reviewers were resolved through discussion.

### Data analysis

Statistical analyses were performed using STATA version 17 (StataCorp LLC, College Station, TX). A random-effects model based on the Sidik-Jonkman method was used for all analyses, regardless of heterogeneity levels. For dichotomous outcomes, odds ratios (ORs) were calculated, while continuous variables were assessed using mean differences. Heterogeneity across studies was evaluated using the I^2^ statistic; values exceeding 50% or a *P* value below .10 was indicative of significant heterogeneity. Meta-regression using the restricted maximum likelihood approach was conducted to explore the impact of baseline and clinical characteristics on arrhythmia recurrence across the study groups. Individual patient-level data were digitally extracted from published Kaplan-Meier curves using Engauge Digitizer and subsequently processed with the “ipdfc” module, thereby enabling the reconstruction of survival analyses and the performance of Cox proportional hazards regression.^7^^,^^8^ The data extraction and reconstruction process involved the identification and extraction of raw coordinate data—corresponding to time points and associated survival probabilities—from the Kaplan-Meier curves. These extracted coordinates were then used to reconstruct IPD in the form of (time, status) pairs, which serve as the foundational structure for survival analysis. The accuracy and consistency of the reconstructed Kaplan-Meier curves were validated through visual comparison with the original published curves for each individual study. After validation, the reconstructed IPD from each study were integrated to generate a comprehensive data set for the evaluation of ATa recurrence. Statistical significance between comparison groups was assessed using the log-rank test, and Cox proportional hazards regression analysis was performed to estimate the hazard ratio with corresponding confidence intervals (CIs). Sensitivity analyses were also performed to evaluate the robustness of the results and to determine the influence of individual studies, particularly those contributing high heterogeneity. Finally, publication bias was assessed using Egger’s test and the funnel plot test followed by trim-and-fill analysis. All statistical tests were 2-tailed, with significance defined as a *P* < .05.

## Results

Eight studies, involving a total of 1411 patients, were included in this systematic review and meta-analysis (Figure 1).^9^, ^10^, ^11^, ^12^, ^13^, ^14^, ^15^, ^16^ The baseline characteristics of the included studies are presented in Table 1. The included studies have a low risk of bias (Table 1).

**Table 1:** Baseline characteristics of the included studies

Study	Design	Sample size	Inclusion	Control group	Paroxysmal AF	Male sex	Age	CHA_2_DS_2_-VASc score	Hypertension	LA diameter	LVEF	3D EAM in HPSD ablation	Additional ablation	Follow-up	NOS score
Badertscher and colleagues^16^	Prospective study (research letter)	115 (52 vs 63)	First PVI	50 W	56%	NA	65 y	NA	NA	41 mm	56%	Pentaspline catheter (Penta Ray)	None	214 d	NA
Popa and colleagues^9^	Retrospective study	127 (35 vs 92)	First PVI	90 and 70 W	100%	58%	63 y	2	50%	NA	59%	Advisor circular catheter	None	6 mo	7
Reinsch and colleagues^10^ (PRIORI)]	Retrospective study	411 (201 vs 210)	First PVI	45 W	100%	55%	68 y	2	67%	NA	NA	Pentaspline catheter (Penta Ray)	None	12 mo	7
Dello Russo and colleagues^11^	PS matched retrospective analysis of the prospective study	342 (171 vs 171)	First/repeat ablation	90 W	68%	64%	66 y	2	62%	NA	60%	Multielectrode mapping catheter	PW ablation, CTI line, LA appendage, others	12 mo	7
Santos and colleagues^12^	Retrospective study	101 (56 vs 45)	First/repeat ablation	90 W	56%	75%	51 y	2	63%	NA	59%	Lasso/pentaspline catheter (Penta Ray)	PW ablation	384 d	8
Soubh and colleagues^13^	Retrospective study	82 (52 vs 30)	First PVI	90 W	46%	62%	69y	4	83%	NA	50%	Pentaspline catheter (Penta Ray)	CTI and LA ablation, especially in the HPSD ablation group	6 mo	8
Weidlich and colleagues^14^	Not described (abstract only)	119 (56 vs 63)	Undergoing PVI	Wattage not described	55%	NA	66 y	NA	NA	41 mm	55%	Multipolar catheter in 47%	NA	177 d	NA
Wörmann and colleagues^15^	Retrospective study	114 (57 vs 57)	First PVI	70 W	30%	37%	67 y	3	63%	39 mm	56%	Circumferential decapolar catheter	None	12 mo	8

3D = 3-dimensional; AF = atrial fibrillation; CTI = cavotricuspid isthmus; EAM = electroanatomic mapping; HPSD = high-power short duration; LA = left atrial; LVEF = left ventricular ejection fraction; NA = not available; NOS = Newcastle-Ottawa Scale; PS = propensity score; PVI = pulmonary vein isolation; PW = posterior wall.

### ATa recurrence

**Figure 2:**
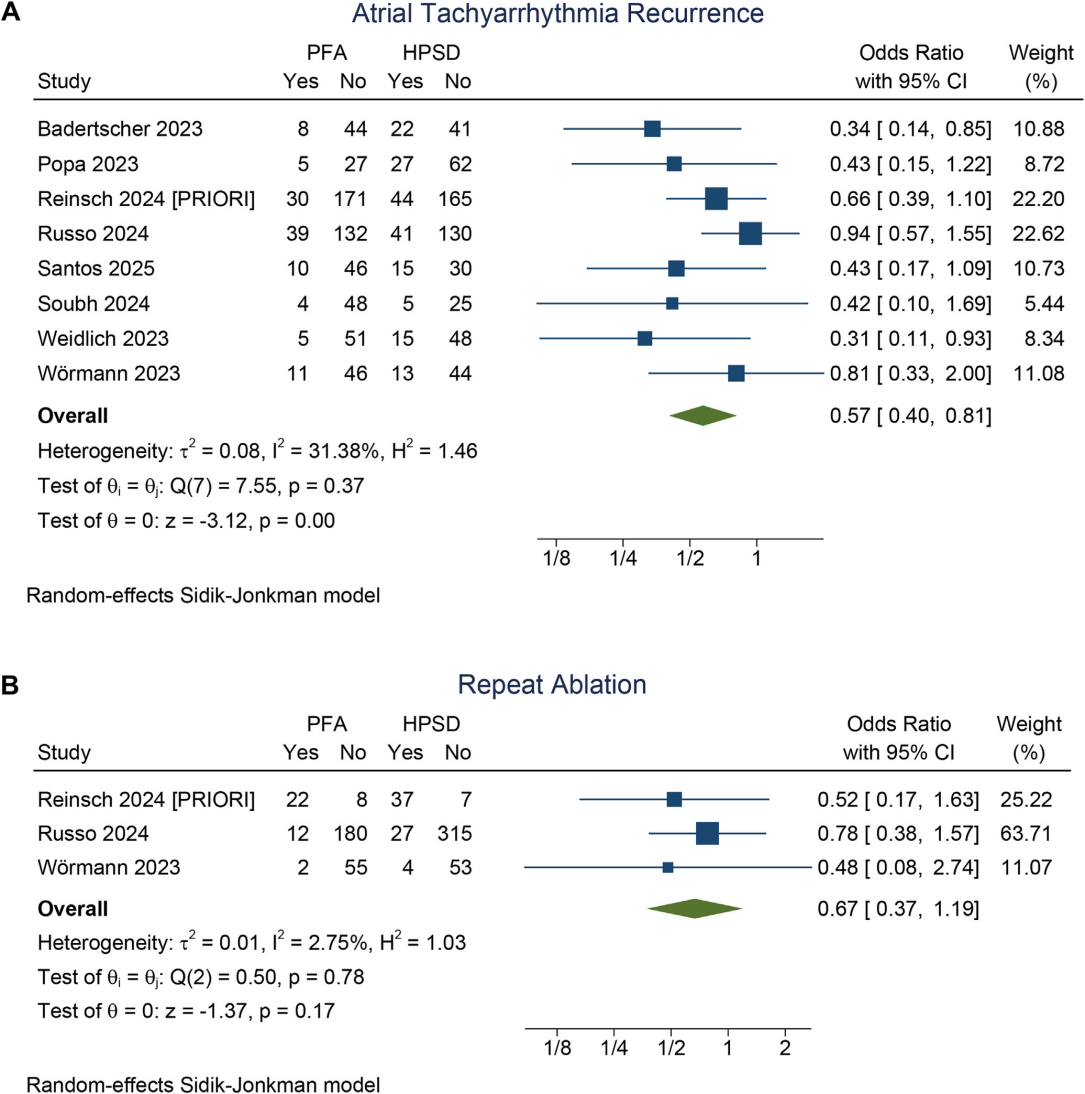
Atrial tachyarrhythmia recurrence (**A**) and repeat ablation (**B**) in pulsed field ablation (PFA) compared with high-power short duration (HPSD)/very high-power short-duration ablation. CI = confidence interval.

The incidence of ATa recurrence was 15% (95% CI 11%–19%) in the PFA group and 25% (95% CI 21%–29%) in the HPSD/VHPSD ablation group. PFA was associated with a significantly lower risk of ATa recurrence compared with HPSD/VHPSD ablation (OR 0.57; 95% CI 0.40–0.81; *P* = .002; I^2^ = 31.4%, *P* = .374) (Figure 2A). A leave-one-out sensitivity analysis showed that the result remained significant (*P* < .05). No significant difference was observed between the 2 groups with regard to the number of repeat ablation procedures (OR 0.67; 95% CI 0.37–1.19; *P* = .172; I^2^ = 2.75%, *P* = .779) (Figure 2B).

### Pulmonary vein reconnection

Pulmonary vein reconnection was reported in 32% (95% CI 24%–39%) of patients in the PFA group and 35% (95% CI 30%–41%) in the HPSD/VHPSD ablation group. The difference in reconnection rates between the groups was not statistically significant (OR 0.84; 95% CI 0.53–1.34; *P* = .473; I^2^ = 6.56%, *P* = .628).

### Reconstructed individual patient data analysis

**Figure 3:**
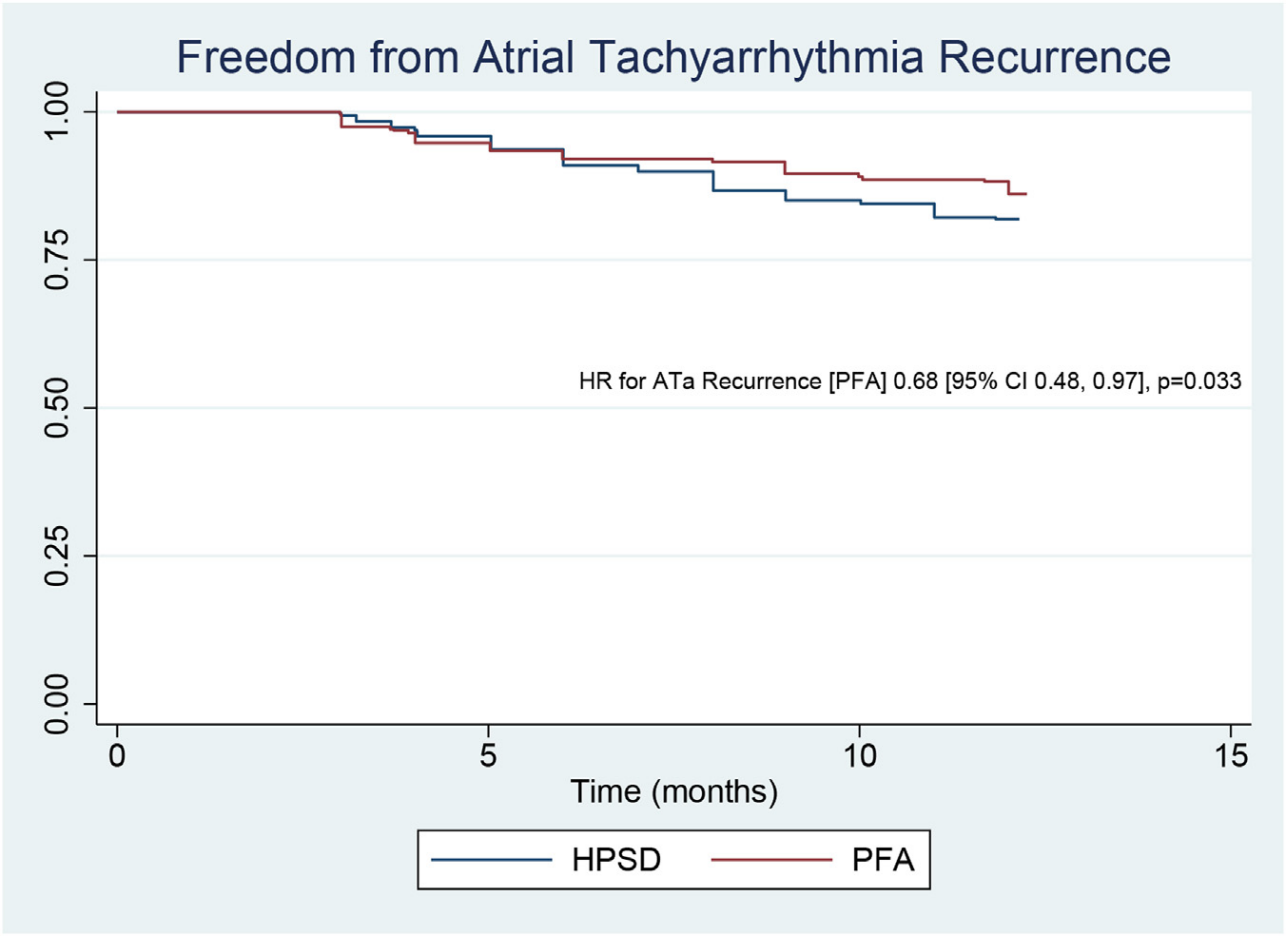
Reconstructed Kaplan-Meier curves and Cox regression analysis comparing atrial tachyarrhythmia (ATa) recurrence between pulsed field ablation (PFA) and high-power short duration (HPSD)/very high-power short-duration ablation. CI = confidence interval; HR = hazard ratio.

Six studies reported Kaplan-Meier curves; their data were processed and combined. The reconstructed Kaplan-Meier curves from these studies indicated a higher rate of freedom from ATa recurrence in the PFA group (Figure 3).^10^, ^11^, ^12^, ^13^^,^^15^^,^^16^ Cox regression analysis based on reconstructed individual patient-level data indicated that PFA was associated with a significantly reduced ATa recurrence (hazard ratio 0.68; 95% CI 0.48–0.97; *P* = .033).

### Procedural duration and fluoroscopy time

**Figure 4:**
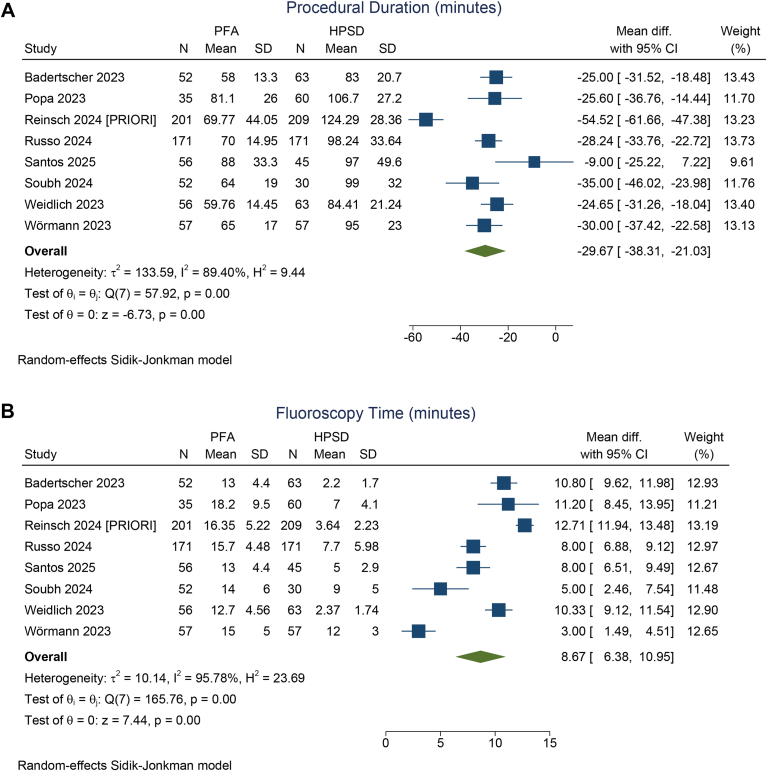
Procedural duration (**A**) and fluoroscopy time (**B**) in pulsed field ablation (PFA) compared with high-power short duration (HPSD)/very high-power short-duration ablation. CI = confidence interval; SD = standard deviation.

PFA was associated with a significantly shorter procedural duration compared with HPSD/VHPSD ablation, with a mean difference of –29.67 minutes (95% CI –38.31 to –21.03 minutes; *P* < .001; I^2^ = 89.4%, *P* < .001) (Figure 4A). Conversely, fluoroscopy time was significantly longer in the PFA group, with a mean difference of 8.66 minutes (95% CI 6.38–10.95 minutes; *P* < .001; I^2^ = 95.8%, *P* < .001) (Figure 4B).

### Complications

The rate of complications was 3% (95% CI 1%–5%) in the PFA group and 4% (95% CI 2%–6%) in the HPSD/VHPSD ablation group; there was no significant difference between the 2 groups (OR 0.94; 95% CI 0.44–2.03; *P* = .342; I^2^ = 33%, *P* = .883). Most complications were related to vascular access; however, cardiac tamponades were observed in 0%–3.5% in the PFA group and 0%–2.8% in the HPSD/VHPSD ablation group (Table 2).

**Table 2:** Reported complications in the included studies

Study	Complications
Badertscher and colleagues^16^	PFA (2) + HPSD ablation (1): 2 tamponades and 1 transient ST-segment elevation
Popa 2023	PFA: Groin complications (5; 14.3%), TIA (1; 2.9%), stroke (1; 2.9%) HPSD ablation: Groin complications (7; 20%), TIA (1; 1.08%)
Reinsch and colleagues^10^ (PRIORI)	PFA: Access site (1; 0.5%), tamponade (3; 1.4%), stroke/TIA (2; 1%) HPSD ablation: Access site (3; 1.4%), tamponade (6; 2.8%), stroke/TIA (2; 0.9%), air embolism (2; 0.9%)
Dello Russo and colleagues^11^	PFA: Pericarditis (1; 1%), femoral arteriovenous fistula/pseudoaneurysm (3; 2%), groin hematoma (1; 1%) VHPSD ablation: Pericarditis (5; 3%), critical limb ischemia requiring angioplasty (1; 1%)
Santos and colleagues^12^	PFA: Tamponade (1; 1.8%) HPSD ablation: None
Soubh and colleagues^13^	PFA: None VHPSD ablation: Stroke/TIA (1; 3%)
Weidlich and colleagues^14^	PFA: Tamponade (1; 1.7%) HPSD ablation: Tamponade (1; 1.6%)
Wörmann and colleagues^15^	PFA: Tamponade (2; 3.5%) VHPSD ablation: Bleeding (2; 3.5%), pulmonary vein stenosis (1; 1.8%)

HPSD = high-power short duration; PFA = pulsed field ablation; TIA = transient ischemic attack; VHPSD = very high-power short duration.

### Meta-regression

Meta-regression analysis demonstrated that the benefit of PFA in reducing ATa recurrence was not significantly influenced by paroxysmal AF (*P* = .861), male sex (*P* = .623), age (*P* = .410), hypertension (*P* = .941), or LVEF (*P* = .283).

### Publication bias

**Figure 5:**
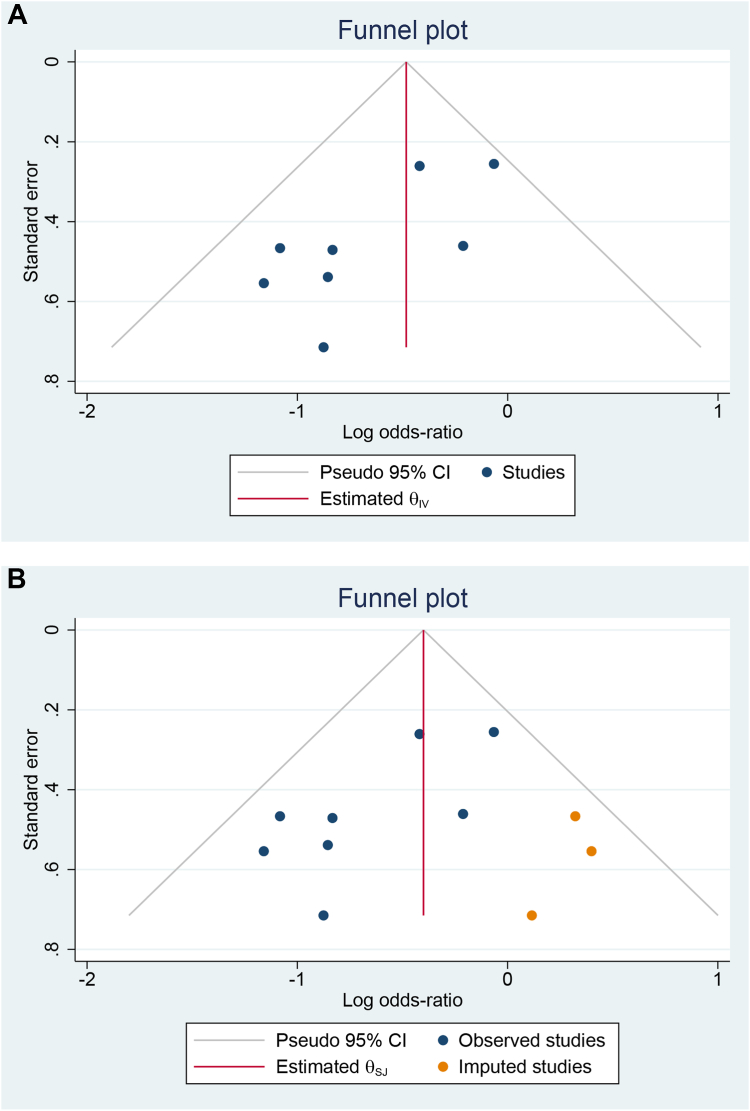
Funnel plot (**A**) and trim-and-fill funnel plot analysis (**B**) comparing atrial tachyarrhythmia recurrence between pulsed field ablation and high-power short duration/very high-power short-duration ablation. CI = confidence interval; θ_IV_ = inverse-variance–weighted effect size; θ_SJ_ = Sidik-Jonkman–adjusted effect size.

Egger’s test did not reveal significant small-study effects with regard to ATa recurrence (*P* = .072). The funnel plot was asymmetrical (Figure 5A), suggesting the possibility of publication bias. Trim-and-fill analysis (L0, imputation to the right side) (Figure 5B) showed that the lower risk of ATa recurrence remained (OR 0.67; 95% CI 0.46–0.97).

## Discussion

This systematic review and meta-analysis indicated that PFA was associated with a lower recurrence rate of ATa compared with HPSD/VHPSD ablation. No significant differences were observed in the number of repeat ablation procedures or in the incidence of pulmonary vein reconnections during repeat procedures. The advantage of PFA in reducing ATa recurrence was not significantly affected by the presence of PAF, sex, age, hypertension, or LVEF. The total procedural duration was shorter in the PFA group, but the fluoroscopy time was longer compared with the HPSD/VHPSD ablation group. Complication rates were similar between the 2 groups.

Reconstructed Kaplan-Meier curves demonstrated that ATa recurrence rates were similar between the 2 groups up to the seventh month. After this point, the curves began to diverge, favoring PFA. In addition, Cox regression analysis based on reconstructed individual patient-level data supported the pooled OR, indicating that PFA was associated with a lower rate of ATa recurrence compared with HPSD/VHPSD ablation. PVI durability appeared to be greater with PFA than with thermal ablation, potentially because of the more transmural lesions produced by PFA, which may reduce the likelihood of pulmonary vein reconnection; however, this was not supported by the present study.^3^ Pulmonary vein reconnection was observed in 32% of patients in the PFA group and 35% in the HPSD ablation group who underwent remapping; however, the difference between the 2 groups was not statistically significant. Reinsch and colleagues^10^ identified the left superior pulmonary vein as the most common site of reconnection in both the PFA and HPSD ablation groups. In contrast, Dello Russo and colleagues^11^ found that the most frequently reconnected vein was the right inferior pulmonary vein in the PFA group and the left superior pulmonary vein in the VHPSD ablation group. However, it remains uncertain whether the use of intracardiac echocardiography to ensure optimal catheter contact during PFA could reduce the rate of pulmonary vein reconnection.

PFA was associated with the loss of acute late gadolinium enhancement during the chronic phase.^3^^,^^17^ In thermal ablation, the breakdown of the structural matrix exposes fibroblasts to mechanical stress, leading to persistent fibrosis. In contrast, the preservation of the extracellular matrix framework after PFA may protect fibroblasts from such stress, thereby maintaining tissue compliance.^3^^,^^17^^,^^18^ This characteristic may offer clinical advantages; however, the included studies did not evaluate echocardiographic parameters of LA function, such as LA strain.

Santos and colleagues^12^ reported that ATa recurrence occurred in 80% of patients in the HPSD ablation group, compared with only 25% in those treated with PFA, suggesting that posterior wall isolation may be more effective with PFA than with HPSD ablation. Tohoku and colleagues^19^ observed that atrial tachycardia was unexpectedly more frequent after PFA than with other ablation modalities. They found that the arrhythmogenic substrate was often related to postablation macroreentrant circuits predominantly located in the posterior wall of the LA, supporting the mechanistic rationale for posterior wall isolation in PFA. However, Dello Russo and colleagues^11^ identified posterior wall reconnection in all patients who underwent posterior wall isolation (2 treated with PFA and 4 with VHPSD ablation). Posterior wall isolation during PFA may be effective and does not seem to prolong procedural duration. Nevertheless, the need for a higher number of PFA applications could be a drawback because of the potential risk of hemolysis.^20^ In contrast, posterior wall isolation appears to be ineffective when using thermal ablation techniques.^21^

The procedural duration was significantly shorter in the PFA group than in the HPSD/VHPSD ablation group, which was expected because of the single-shot nature of the PFA system. However, the mean differences in procedural duration and fluoroscopy time across studies were highly heterogeneous. This variability likely stems from the control group’s composition, which included both HPSD and VHPSD ablation techniques—where VHPSD ablation may allow faster procedures. Mapping catheters used might also affect procedural duration. Moreover, since PFA is a relatively new technology, the learning curve could have contributed significantly to both procedural and fluoroscopy times.^12^ Additional ablation beyond PVI in the HPSD/VHPSD ablation group also extended procedural time. In contrast, LA posterior wall isolation can be performed without notably increasing either procedural duration or fluoroscopy time.^20^ Furthermore, operators experienced in thermal ablation may rely more heavily on 3-dimensional electroanatomic mapping and require minimal fluoroscopy. Conversely, PFA procedures often necessitate fluoroscopy to confirm catheter rotation and positioning.^12^

### Limitations

A key limitation of this study is the absence of randomized controlled trials, with most included studies being retrospective in nature. In addition, follow-up durations were relatively short, highlighting the need for more studies with follow-up periods beyond 12 months. However, this limitation is understandable, given that PFA is a relatively new technology. Furthermore, more detailed and standardized reporting of prognostic variables, such as LA diameter, is necessary to enable more robust meta-regression analyses. Further large randomized controlled trials were required to confirm these findings. In addition, pulmonary vein reconnection mapping was conducted in patients who underwent repeat procedures, as determined through shared decision making between the physician and the patient. Further randomized controlled trials are required to provide prospective, long-term, head-to-head comparisons between PFA and HPSD/VHPSD ablation with respect to ATa recurrence and complication rates. In addition, future studies should aim to identify and report risk factors associated with ATa recurrence to evaluate their impact on outcomes and to determine whether specific patient subgroups may derive greater benefit from one ablation strategy over the other.

## Conclusion

PFA was potentially associated with a comparable or reduced rate of ATa recurrence compared with HPSD/VHPSD ablation while maintaining comparable complication rates. However, this observation should be interpreted with caution owing to the observational nature of the included studies. Further randomized controlled trials are warranted to validate these findings. No significant differences were observed in the number of repeat ablation procedures or the incidence of pulmonary vein reconnections during repeat procedures. The total procedural duration was shorter in the PFA group, but the fluoroscopy time was longer compared with the HPSD/VHPSD ablation group.
